# Localized doxycycline delivery via polymeric nanoparticles anchored to electrospun PCL microfiber patch for MMP-2 inhibition in abdominal aortic aneurysm

**DOI:** 10.1007/s13346-025-02027-z

**Published:** 2026-01-20

**Authors:** Carles Bofill-Bonet, Germán Febas, Margalida Artigues, Inés Moreno-Jiménez, Noemí Balà, Jordi Martorell, Salvador Borrós, Cristina Fornaguera

**Affiliations:** 1 Institut Químic de Sarrià (IQS), Universitat Ramon Llull (URL), Via Augusta 390, 08017 Barcelona, Spain; 2 Aortyx SL, Teia, Spain

**Keywords:** Water-In-Oil-In-Water nano-emulsions, Polymeric nanoparticles, Doxycycline encapsulation, Abdominal aortic aneurysm therapies, Nanoparticle anchored nanofibers

## Abstract

**Abstract:**

Doxycycline (DOXY) is a well-established antibiotic that has recently shown potential in inhibiting matrix metalloproteinase-2 (MMP-2), a key enzyme involved in the progression of abdominal aortic aneurysms (AAA). However, the controlled delivery of DOXY to the aneurysm site, with sustained release and minimal systemic exposure, remains a critical challenge in therapeutic development. To address this, we developed a targeted drug delivery platform based on polymeric nanoparticles (NPs), prepared from water-in-oil-in-water nano-emulsions, encapsulating DOXY and are covalently attached to electrospun ε-poly(caprolactone) (ε-PCL) microfibers. This system was designed to enable local, sustained drug release in the inner wall of aorta while preserving the mechanical properties of the aortic wall. The ε-PCL electrospun microfibers from the patch were first functionalized using oxygen cold plasma treatment, creating free radicals that enabled covalent bonding with chemical groups on the outer layer of DOXY-loaded poly(lactic-co-glycolic acid) (PLGA) NPs. This strategy allowed for robust immobilization of the NPs onto the microfibers surface, forming a composite system capable of localized and controlled drug release over time. Unlike traditional delivery approaches, this method ensures site-specific action of DOXY directly at the aneurysmal tissue, minimizing systemic circulation and reducing off-target toxicity. The platform not only provides a stable drug reservoir but also offers intrinsic biomechanical reinforcement, which is critical in AAA condition. This innovative delivery system represents a significant advance in the localized treatment of vascular disorders. It offers a biocompatible, biodegradable, and precisely targeted therapeutic approach, with potential to reduce the need for surgical intervention and limits the adverse effects associated with systemic drug administration.

**Highlights:**

- Novel polymeric Doxycycline loaded PLGA nanoparticles have been developed and result efficacious within hMMP-2 mitigation and collagen degradation in Abdominal Aortic Aneurysm condition.

- Doxycycline loaded polymeric nanoparticles were covalently anchored to ε-Poly(caprolactone) electrospun microfibers via cold plasma-induced radical grafting, enabling sustained drug release for over 12 days.

- Doxycycline-loaded nanoparticles released from microfibers efficaciously mitigate hMMP-2 in human in vitro models of Abdominal Aortic Aneurysm.

- Doxycycline released from drug-coated electrospun ε-Poly(caprolactone) although efficacious does not allows time-control.

**Graphical Abstract:**

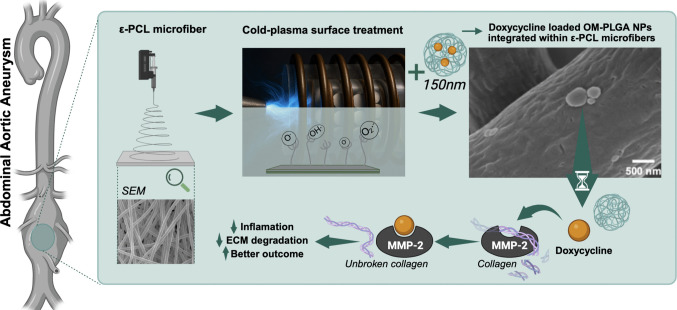

**Supplementary Information:**

The online version contains supplementary material available at 10.1007/s13346-025-02027-z.

## Introduction

Vascular remodeling plays a critical role in maintaining the structural integrity of the aortic wall, facilitating adaptation to mechanical and chemical changes and maintaining homeostasis. This dynamic process orchestrates cellular activities such as growth, apoptosis, migration, and the synthesis or breakdown of the extracellular matrix (ECM) [1]. Central to ECM turnover are matrix metalloproteinases (MMPs), a family of 23 types of enzymes responsible for degrading proteins, especially collagen (MMP-2 and −9), elastin and other ECM components to create space for tissue regeneration and repair. However, dysregulation of MMP activity, often triggered by environmental factors or genetic predispositions, can lead to excessive ECM degradation, contributing to pathologies such as Abdominal Aortic Aneurysm (AAA) [2].

AAA, first documented in 1555, manifests as a pathological dilation of the abdominal aorta exceeding its normal size (> 50%), accompanied by ECM mechanical property loss [3]. Enhanced MMP activity and reduced MMP inhibition leads to ECM over-degradation, preventing the aorta from returning to its original shape during blood flow pressure variations and predisposing it to rupture or dissection. The consequences of AAA rupture are dire, often resulting in internal hemorrhage or compression of adjacent organs, leading to ischemia or further rupture, with mortality rates reaching alarming levels [4, 5]. Although predominantly affecting older men with associated cardiovascular risk factors, AAA has a multifactorial etiology, including genetic predispositions such as Marfan or Ehlers-Danlos syndromes. Despite its significant global incidence and mortality rates, AAA often progresses silently until reaching advanced stages, emphasizing the urgent need for effective interventions [6].

Current management strategies for AAA primarily focus on blood pressure control and symptomatic management for non-severe aneurysms and the use of endovascular repair to place a stent graft that prevents aortic burst. However, these approaches do not address the underlying MMP dysregulation or promote aortic wall regeneration. Moreover, they have long-term negative impacts such as endoleaks and sac growth leading to re-intervention over time [7]. Encouragingly, recent studies highlight the potential of doxycycline (DOXY), a broad-spectrum antibiotic, in reversing MMP-mediated pathology in AAA through MMP-2 inhibition. Nevertheless, systemic administration of DOXY poses challenges due to poor absorption in the abdominal aorta and associated adverse and off-target effects [8].

Nanotechnology could overcome these limitations by offering targeted drug delivery and sustained controlled release only at the damage site. Non-viral polymeric nanoparticles (NPs) present an attractive platform for local DOXY delivery, providing time-controlled release and minimizing systemic side effects [8].

To ensure the targeted delivery of NPs to the damaged tissue, several strategies can be employed, including NPs surface functionalization with targeting ligands. For instance, Dosta et al. demonstrated efficient targeting by conjugating polymeric NPs with a VCAM-1-binding peptide, which significantly enhanced transfection efficiency in VCAM-expressing cells [9].

While such ligand-based strategies offer high specificity and transfection rates, they pose considerable challenges in highly vascularized regions such as the aorta, where nonspecific distribution and rapid clearance may compromise efficacy. To overcome these limitations, we propose here an alternative approach based on local, non-targeted administration. Specifically, NPs were embedded within biodegradable polymeric microfibers, forming a patch designed to serve as both a localized drug delivery platform and a biomimetic scaffold. These microfibers not only facilitate sustained and spatially confined release of the therapeutic payload, but also provide biomechanical advantages, such as dynamic compliance with the natural pulsatile movement of the aortic wall as reported before by Balà et al. [10].

Advances in nanotechnology open new and powerful ways to tackle the unmet challenges in treating AAA. By targeting MMP dysregulation and promoting aortic wall regeneration, our innovative intervention paves the way for effective, minimally invasive treatments for AAA and move towards precision medicine.

## Experimental section

### Materials

Doxycycline (DOXY) Hyclate (CAS: 24390–14-5, C22H24N2O8·HCl·0.5H2O· 0.5C2H6O), 93.5% of purity) and PLGA Resomer 502 (acid terminated, MW 7000–17000 g/mol, 50:50 LA:GA ratio), Pluronic F-127 and Tween 80 (cel9l culture suitable, 98% of purity) were purchased in Sigma Aldrich. (H-Cys-Arg-Arg-Arg-NH2) oligopeptides were obtained from Genecust (France) with > 98% purity. ELISA assay kit with, were provided by Abcam. Other products were purchased in ThermoFischer. Anti-collagen Type I, rabbit and Anti-Goat IgG H&L (Alexa Fluor® 488) were purchased in Abcam.

### Cell lines

Human Aortic Smooth Muscle cells (referred to as HAoSMC) from Promo Cell (ATCC PCS-100-012TM) were used. HAoSMC are primary cells directly isolated from human aorta tissues. The cell culture medium, Smooth Muscle Cell Basal Medium from Promo Cell, was supplemented with 5% (v/v) fetal calf serum (FCS), 1100 IU/ml penicillin/streptomycin and Smooth Muscle Cell Supplement Pack GM (containing Epidermal Growth Factor, basic Fibroblast Growth Factor, and insulin). Cell cultures were maintained at 37 °C in a humidified atmosphere with 5% CO2 and 95% air.

### Methods

#### Doxycycline dosing regimens

To compare the effect of different doxycycline administration strategies, two dosing regimens with equivalent total DOXY exposure were applied to the AAA in-vitro models. In the *single-dose condition*, cells received a single administration of DOXY at a final concentration of 80.0 µM. In the *cumulative-dose* condition, cells received four sequential administrations of DOXY (20.0 µM each), delivered at equal time intervals, yielding the same cumulative concentration (80.0 µM). Following treatment, the different dosing strategies were evaluated in terms of cell viability, total MMP activity and MMP-2 secretion as further detailed in Sects. "Viability cellular test", "Gelatin zymography" and "MMP-2 ELISA", respectively.

#### ε-PCL formulation to produce microfiber sheets by electrospinning

Prior to electrospinning, the initial step is to prepare a PCL solution using a mixture of organic solvents. This is accomplished by ε-PCL-17 10% w/v solution in a mixture of chloroform and methanol (95:5) ratio. The entire solution is then sealed within a 30 mL flask, with constant stirring (150 rpm). The viscosity of each polymer organic solution was determined utilizing a rheometer equipped with a cone-plate configuration (Physica MCR 501, Anton Paar). The measuring cone, designated as CP50-1, had a diameter of 50 mm and an angle of 1 degree. The temperature of both the cone and the plate was maintained at 20.0 °C for all the solutions. Briefly, 2 mL were placed on the rheometer plate and evaluated with a continuous shear rate (1/s) ramp and evaluating shear stress (Pa). Then the viscosity slope was plotted as mean with standard error.

To obtain electrospun microfibers the previous polymeric organic solution is loaded into a syringe and placed in Fluidnatek LE-10 (Bioinica) equipped with a needle of 0.6 mm and connected to the nozzle. The fibers were deposited using a 200 Ω conductive foil placed in a 10 cm diameter drum. To obtain a good fibbers deposition ratio and desired morphology, flow rate, voltage and collector speed was selected according to the defined specifications (see ESI-1).

#### Microfiber morphology evaluation by scanning electron microscopy

A small triangular-shaped sample was cut from the manufactured sheet, placed in carbon tape, coated with gold, and then positioned in the IQS SEM (Jeol JSM) 5310. 40 fibers were selected randomly to quantify the diameter distribution and morphology using imageJ software.

#### ε-Polycaprolactone microfibers sheet hydrophobicity calculation

To evaluate the hydrophobicity of the material, DSA (KRUS) equipment was used. 10 pieces of 10 mm × 5 mm were analyzed measuring the contact angle using 100 µL droplet of MiliQ water.

#### Doxycycline quantification

To quantify the drug content within the fibers, a 2 cm^2^ section was cut and weighed. Samples were dissolved in chloroform (5.0 mL), followed by three liquid–liquid extractions using 2.0 mL of Milli-Q water. The aqueous phases, filtered with a Nylon 0.22 µm filters, were combined and analyzed for doxycycline content using by ExionLC™ AD UHPLC system coupled to SCIEX Triple Quad™ 7500 MS/MS – QTRAP®. For release profiling, 2 cm^2^ fiber patches were weighed and immersed in 30.0 mL of PBS (pH 7.4) in glass vials, incubated at 37 °C under constant agitation (200 rpm). At each timepoint, the full release medium was collected and replaced. An aliquot (1 mL) was filtered with a Nylon 0.22 µm filters diluted (1:10) and analyzed by ExionLC™ AD UHPLC system coupled to SCIEX Triple Quad™ 7500 MS/MS – QTRAP®.

#### Analytical method for doxycycline and degradation products

Column ACQUITY UPLC BEH C18 1.7 µm, 2.1 × 100 mm was used. The mobile phases were: (A) Milli-Q water with 0.1% formic acid (pH 1), and (B) acetonitrile. 1 min at 5% B; 1–7 min, 5–100% B; 7–9 min, 100% B; 9–9.1 min, 100–5% B; 1 min at initial conditions B. Injection volume was 5 μL. ESI source parameters were: + 5500 V spray voltage, 500 °C temperature, 45 psi nebulizer gas, 60 psi auxiliary gase, and 40 psi curtain gas. Data acquisition was performed using a multireaction monitoring (MRM) method following the quantifier transition 445.2 > 428.3 (CE = 25 V) and the qualifier transition 445.2 > 339.2 (CE = 40 V) for DOXY (RT = 4.14 min). Data were acquired and processed with SCIEX OS 3.1.0.16485.

#### Nano-emulsion and NPs formulation

Water_1_-in-oil-in-water_2_ (W_1_/O/W_2_) template emulsions were formulated for nanoparticles formulation as previously reported [11]. In brief W_1_/O [doxycycline in aqueous solution (W_1,_ 20%wv)/1%wv Pluronic F-127® as surfactant (S_1_) and 4%wv PLGA in ethyl acetate (O, 90%wv)] was formed by rotor–stator high speed homogenizer. Both phases (W_1_ and O) were homogenized for 2 min at 30,000 rpm using Ultra-Turrax®. Then emulsion was mixed with Tween 80®, proceeding to prepare O/W_2_ emulsion.

#### Hydrodynamic diameter, polydispersity index and surface charge determination

The mean nano-emulsion droplet and nanoparticle hydrodynamic diameter were determined by DLS, (ZetaSizer NanoZS, from Malvern Co. Ltd., UK), equipped with a He–Ne red light laser ($\lambda$= 633 nm). Measurements were carried out without diluting the samples (85 µL), at a scattering angle of 180º, and under a constant temperature of 25 ºC. 10 runs were performed for each sample. Data was treated by cumulant analysis [11]. Mean size and concentration have been characterized using Nano Tracking Analysis with 1.0 mL of the NPs dispersion diluted 1/100. Nano Sight NS300 from Malvern was used. The samples were measured 5 times for 60 s. A total number of 80–100 NPs per frame were analyzed.

Surface charge was determined in the same manner and equipment. This instrument measures the velocity of the droplets or particles in the electric field (electrophoretic mobility) using the laser Doppler electrophoresis method [12–14]. The zeta potential (ζ) was calculated from the electrophoretic mobility applying the Smoluchowsky Eq [14, 15].

#### Cryo-TEM morphological determination

Cryo transmission electron microscopy (cryoTEM) was used to characterize the size and shape of NPs. A drop of 10 µL was incubated in a carbon-coated copper 200 mesh grid for one minute. Following, grids were loaded into the FEI Vitrobot Mark IV, blotted briefly, and plunged into liquid nitrogen. Frozen grids were transferred to the cryo-holder and observed in Jeol 1400 microscope. The mean size was determined by image analysis with Image J software.

#### Confocal microscopy

For Fig. 6, DOXY-loaded OM-PLGA-Fluorescein Isothiocyanate—PLGA (1:1) NPs were formulated to evaluate uptake by confocal microscopy. After treatment, cells were fixed with 10% formalin (v/v) and permeabilized with 0.1% Triton X-100 (v/v) for 20 min. Coverslips were removed from the well plate and stained with DAPI for nuclear visualization and Red Cell Mask for plasma membrane staining. Samples were imaged using a laser-scanning confocal spectral microscope (Leica SP8) equipped with Argon and He–Ne lasers and coupled to a DMI6000 inverted microscope.

For the assays shown in Fig. 8, HAoSMCs were cultured for 8 days under starvation conditions (0.5% FCS) in the presence of ascorbic acid (50 µg/mL) using confocal gelatin-coated Petri dishes to promote extracellular matrix deposition. After this conditioning period, cells were exposed to the corresponding treatments for 4 days.

Type I collagen was immunostained by incubating the samples with the primary anti-collagen I antibody for 24 h at 4 °C in 3% w/w BSA, followed by incubation with an Alexa Fluor 488-conjugated secondary antibody. Nuclear staining was performed with DAPI, and plasma membranes were labelled using Red Cell Mask prior to confocal imaging. Sample exposure settings have been the same for all conditions.

#### Surface treatment with cold oxygen plasma for NP attachment

Plasma reactor (Mandalino) has been 12 h making vacuum at 1·10–3 mBarr. Subsequently, the microfibers were introduced, and upon achieving consistent vacuum values, the following conditions outlined were implemented: System pressure (2.3 × 10–2 mbar), Oxygen flow rate (9 mL/min), working pressure (1.4 × 10–1 mbar), power (10 V), Pulse ON – Pulse off (1–20 ms), time and cycles (2 × 25″).

#### Field emission scanning electronic microscopy (FESEM)

Fiber samples for FESEM were coated with gold and then examined using the SEM Zeiss Merlin microscope at the Universitat Autònoma de Barcelona. Multiple images were captured. Electron Beam energy was set at 3.00 kV and working distance at 5.2 mm.

#### Viability cellular test

Cell viability was assessed with the 3-(4,5-dimethylthiazol-2-yl)−2,5-diphenyltetrazolium bromide (MTT) colorimetric assay (protocol modified from the GTA-1/2 protocols of the NCL assay cascade) [13].

#### Cell model and sample preparation for MMP-2 quantification

After treatment, supernatants and cell lysates were collected. Cells were lysed with RIPA buffer (1:3 of well volume) containing protease inhibitors, followed by three freeze–thaw cycles and 5 min sonication. All samples were centrifuged at 3000 rpm for 5 min at 4 °C.

#### Gelatin zymography

MMP 2 and 9 activity was assessed by gelatin zymography. Samples (20 μg total protein) were prepared in non-reducing, non-denaturing SDS buffer and loaded onto 10% Novex™ Zymogram Plus gels. Following electrophoresis, gels were renatured and developed using commercial and incubated overnight at 37 °C. Proteolytic bands were visualized with Coomassie staining and quantified using ImageJ.

#### MMP-2 ELISA

MMP-2 concentration was determined using ab100606, Abcam ELISA kit, following manufacturer’s instructions. Absorbance was measured at 450 nm. A standard curve (5–12.000 pg/mL; R^2^ = 0.9922) was used for quantification. Values were normalized to total protein and sample volume.

#### In vivo acute cytotoxicity studies

Acute systemic toxicity, following ISO 10993–11 guidelines (UNE-EN ISO 10993–11:2018), was assessed through intravenous administration. Initially, a preliminary study was conducted to assess feasibility. This involved an acclimation period of 6 days, followed by the intravenous administration of 200 µL of each sample into female *Mus Musculus* (OF1 Ico (IOPS Caw) from Charles River Laboratories, weighing between 17.1 and 20.2 g at the injection timepoint. Each sample was administered twice into two mice, with clinical observations recorded at 4 h, 24 h, 48 h, and 72 h post-injection, along with weight measurements at each time point. Subsequently, a single dose of the tested condition was injected into each mouse. Dosing occurred on day 0. Mice were observed for any adverse clinical reactions immediately after injection. The mice were observed for signs of systemic reactions at 4, 24, 48 and 72 h, and weighed at 24, 48 and 72 h after injection. After the test was completed, all mice were euthanized.

### Statistical analysis

GraphPad Prism software APA was used for graph design and statistical analysis. For all samples, results are given as mean values ± standard deviation of, at least, three independent samples. A non-parametric multiple t-test (Mann–Whitney U test) was used to determine statistical differences between the condition of study and the non-treated control. Results were labeled as * *p* < 0.05, ** *p* < 0.01, and *** *p* < 0.001. Instances where p > 0.05 are denoted as "ns" (non-significant). Results treated with another statistical analysis are reflected in picture captions.

## Results and discussion

### Preliminary determination of free DOXY therapeutic effects on HAoSMCs

**Fig. 1:**
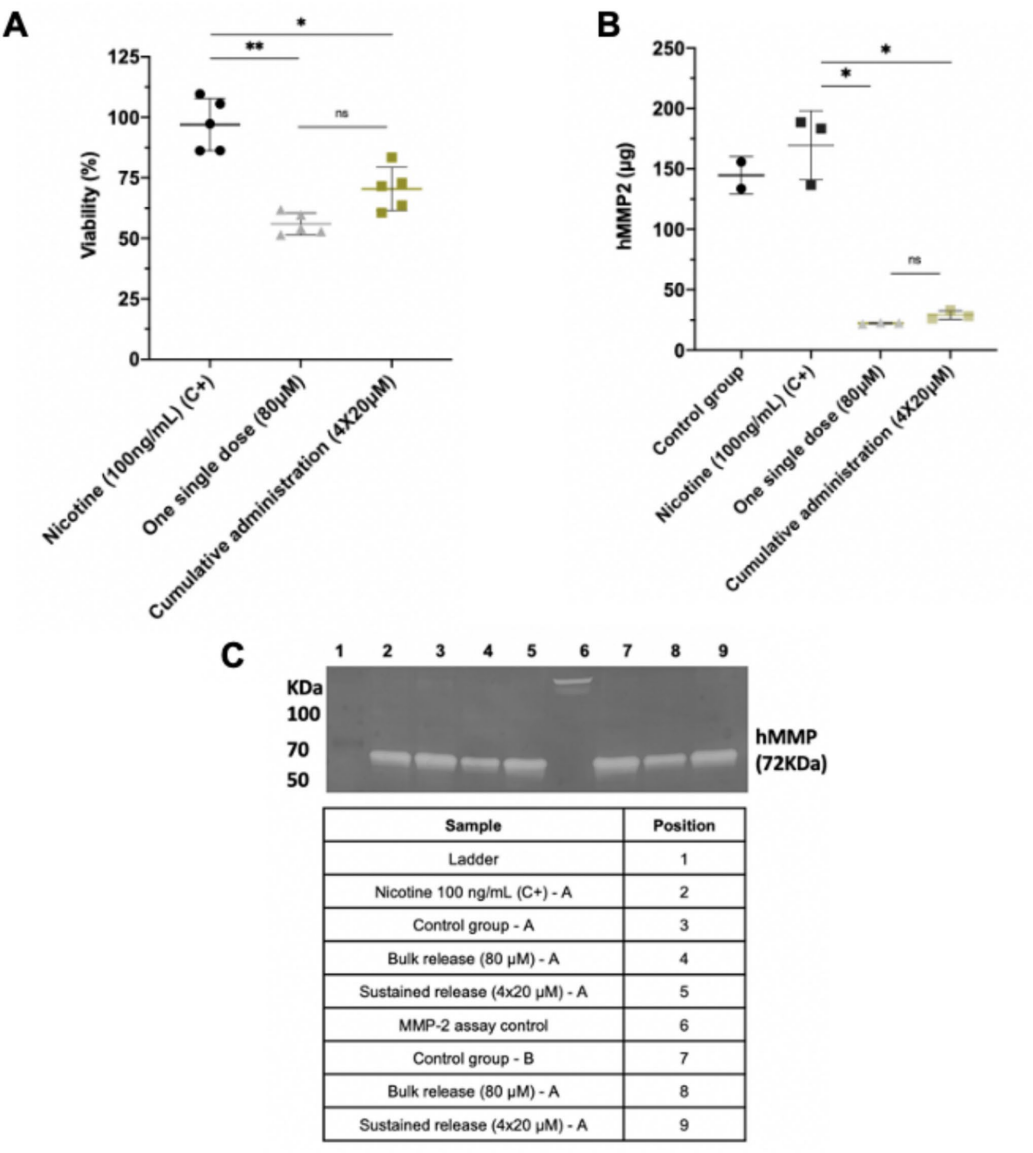
**A**—DOXY cytotoxicity characterization 24 h after exposure in HAoSMC cell line by MTT assay. **B**—Effect of DOXY in AAA in vitro models assessed by MMP-2 concentration quantification through MMP-2 ELISA after 4 days treatment. **C**—Effect of DOXY in AAA in vitro models assessed by MMP-2 activity characterization through zymography assay

The first step in this study was the development of an in vitro model to study the effect of drug treatment on the downregulation of matrix metalloproteinase type 2 (MMP-2). In this context, scientific literature supports the use of nicotine as a stimulus to establish a reliable in vitro model that recapitulates key pathological features of abdominal aortic aneurysm (AAA). Studies by Cerofolini et al*.* and Cabral-Pacheco et al*.* demonstrated that doxycycline (DOXY) effectively reduces the expression of MMP-2 in human aortic smooth muscle cells (HAoSMCs) [8, 16]. In our study, both findings were integrated and experimentally validated to develop a robust in vitro model of AAA, ensuring that the pathological conditions simulated do not interfere with the pharmacological activity of DOXY. Crucially, this model also enabled the systematic evaluation of different administration profiles, aiming to identify the most efficacious therapeutic strategy.. Notably, as demonstrated in Fig. 1A, DOXY cumulative administration significantly reduced cytotoxic effects while maintains drug efficacy, reflected in MMP-2 activity and concentration mitigation (Fig. 1B and Fig. 1C, respectively) when compared with one single-dose administration.

### Stablishig DOXY-embedded microfibers in vitro efficacy

Following the confirmation of free DOXY’s efficacy in downregulating overexpressed MMP-2 in HAoSMCs under AAA conditions, the therapeutic strategy was initiated. As a first step, poly(ε-caprolactone) (PCL) microfibers were synthesized to serve as the base platform for drug delivery forming a patch. To this end, increasing concentrations of DOXY (ranging from 0 to 1.0 mg/gram of organic solution) were incorporated into organic solution prior to the electrospinning process. In this context, DOXY served as a benchmark to evaluate, at a later stage, the relative efficacy of the NP-based delivery system. Electrospinning was conducted under the conditions described (see ESI-1).

**Fig. 2:**
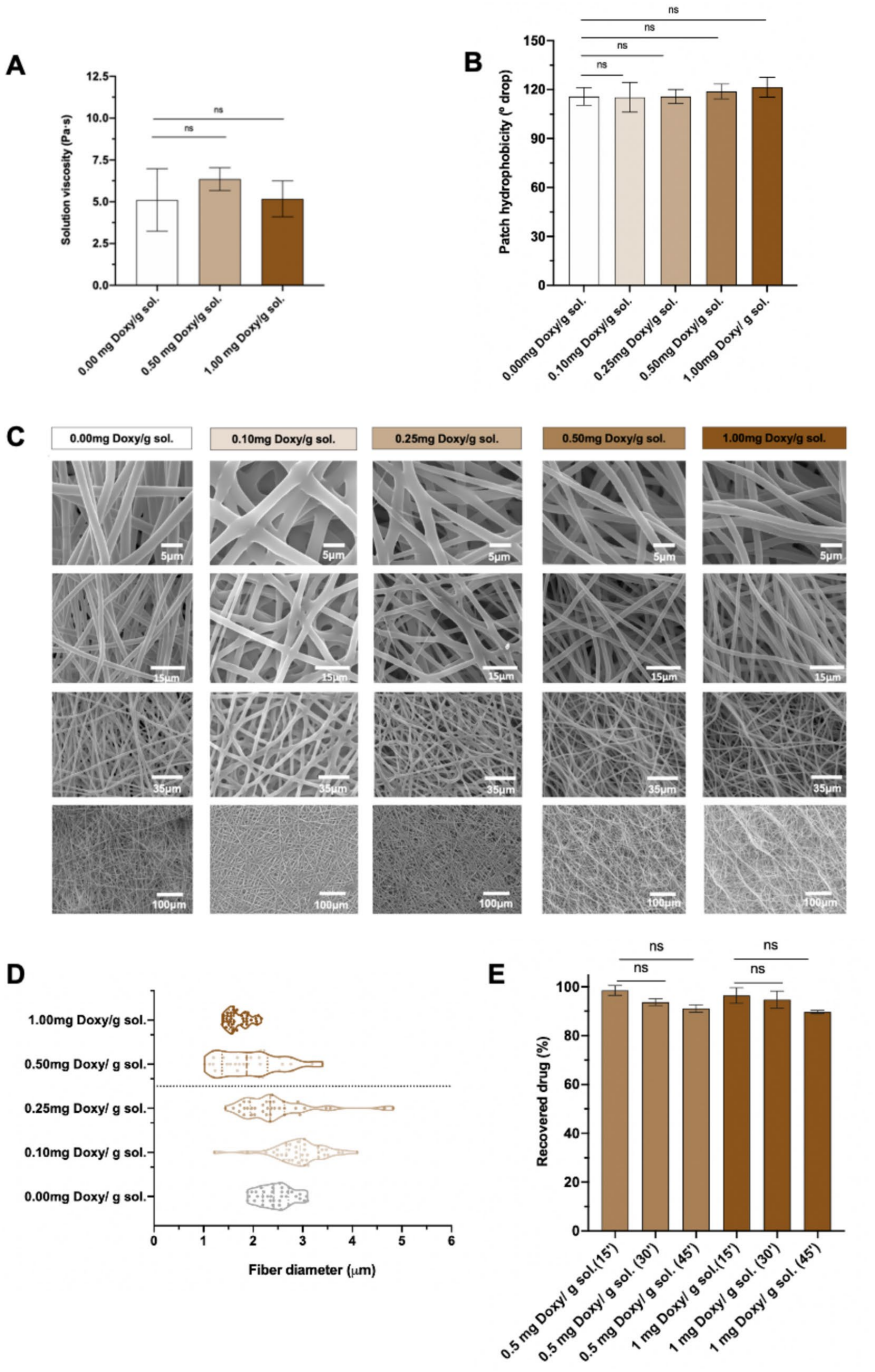
ε-PCL electrospun microfiber characterization. **A** – Viscosity characterization of the different solution through rheological studies. **B** – Microfiber hydrophobicity characterization through Drop Shape Analysis (DSA) (*n* = 10). **C** – SEM images at different magnifications (from left to right 0.5 kX, 1kX, 10kX, 20kX). **D** – Microfiber diameter characterization through Image J quantification of the previous SEM pictures. **E** – DOXY quantification through UHPLC-MS QTrap 7500 after different electrospinning times and concentrations

Initial analyses focused on assessing the drug’s impact on the rheological properties of the polymeric solution, with particular attention to viscosity. As illustrated in Fig. 2A, no significant changes in viscosity were observed with increasing DOXY concentrations, indicating that the solution maintained suitable rheological characteristics for successful electrospinning. These findings are consistent with previously reported data, such as those presented by Balà et al. [10] where microfibers sheets were crafted from a solution with a viscosity of 1.87 ± 0.12 kg/m·s at 20ºC and 200 s^−1^.

A comprehensive analysis was subsequently conducted to evaluate the impact of DOXY incorporation on the morphology and deposition pattern of the electrospun microfibers, examining structural characteristics from the outer to inner layers. Additionally, the hydrophobicity of the resulting microfiber patch was assessed. As shown in Fig. 2B, the presence of DOXY did not significantly affect the hydrophobic properties of the final construct exceeding angle below 115º for all condition. Further morphological analysis (Fig. 2C**)** revealed a consistent random arrangement of micrometric fibers across all conditions, including those with the highest DOXY concentrations. These structures closely resembled the control samples and exhibited no signs of surface roughness. However, quantitative analysis revealed minor yet statistically significant differences in microfiber diameter varying from 1.0 ± 0.2 µm without drug to 1.8 ± 0.3 µm with the maximum amount of drug. Despite these variations, all measured fiber sizes remained within the acceptable range to preserve the functional properties of the patch **(**Fig. 2D**)** similar to the 3 ± 0.5 µm value reported by Balà et al. [10].

To confirm the successful retention of the drug within the fibers following electrospinning, DOXY content was quantified across different drug concentrations and at various time points. The results indicated that only a minimal amount of drug was lost during the electrospinning process (< 10%), confirming the retention of DOXY within microfibers **(**Fig. 2E**)**.

The overall structural integrity of the system remained unaffected, despite minor variations observed in the microfiber deposition pattern and diameter that could be optimized adjusting electrospinning parameters. Compared to previous findings reported by Opálková et al*.* the microfibers generated in this study exhibited greater homogeneity in contrast to observations reported by other authors probably due to greater homogeneity of our polymeric solution enhancing methodologic robustness [17, 18].

**Fig. 3:**
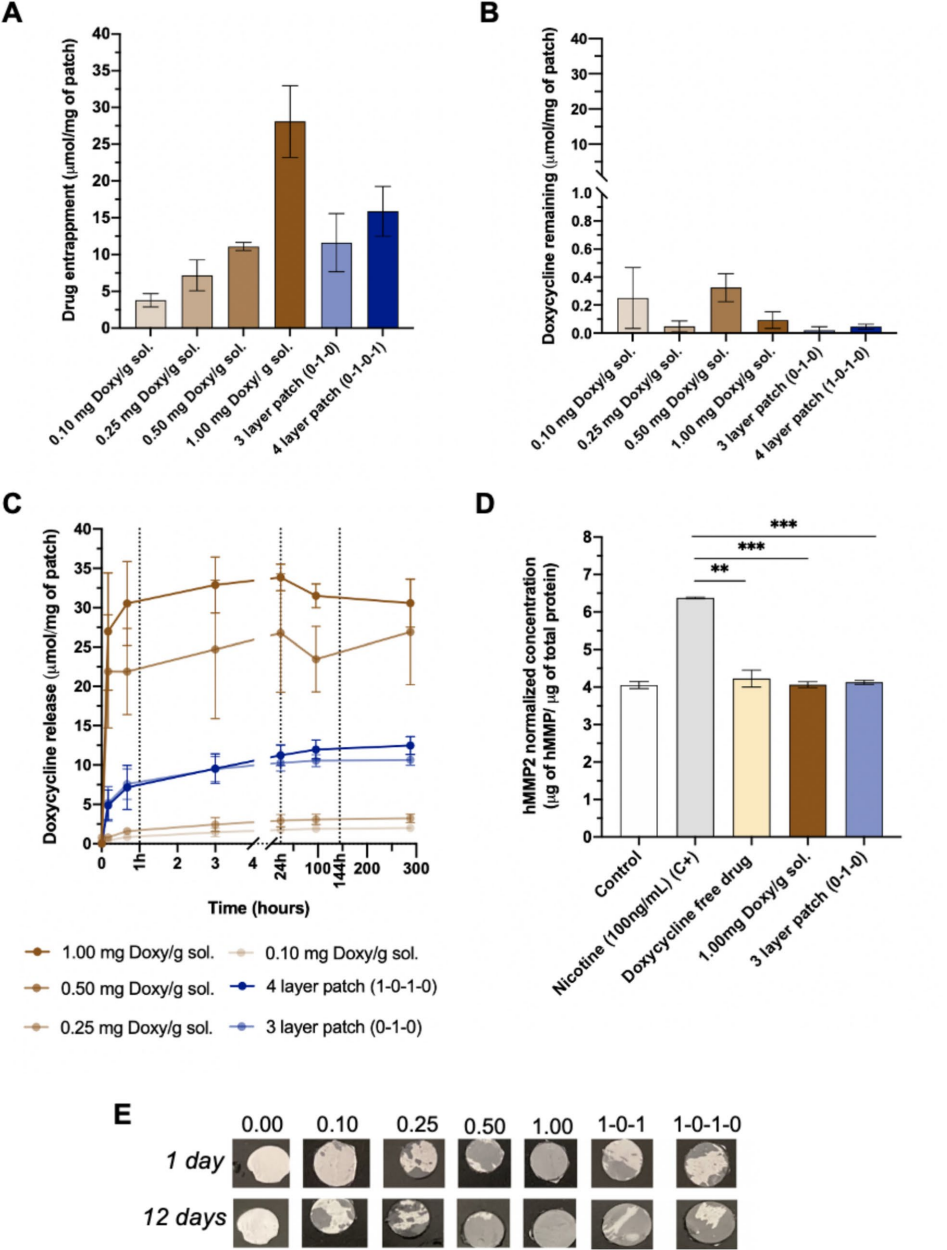
Encapsulation and release profile of DOXY within PCL microfibers. **A** – Quantification of DOXY encapsulated in microfibers as a function of initial drug concentration in the spinning solution, measured by UHPLC–MS. **B** – Residual DOXY content in the microfibers after 12 days of release, corresponding to data shown in panel (**C**). **D** – Evaluation of patch surface wettability over time and with drug loading. As *n *= 6, a parametric unpaired t-test was applied in (**D**)

As expected and shown in Fig. 3A, increasing the drug concentration in the polymeric solution led to a greater amount of DOXY retained within the microfibers following the electrospinning process. Additionally, multilayered sheets composed of alternating drug-loaded (1.00 mg/g of organic solution) and non-loaded layers of microfibers (referred to as 1–0–1 or 1–0–1–0 configurations) were designed to investigate whether the quantified drug was primarily distributed on the fiber surface or embedded within the internal structure.

Following this rationale, the system was exposed to an aqueous solution simulating semi-physiological conditions to characterize the drug release profile of DOXY from the patch. Additionally, the residual drug content in the microfibers was quantified after 12 days of incubation. As shown in Fig. 3B, no DOXY (< 0.5 µmol/mg of patch) remained in the fibers after this period, thus, the whole drug content had been released from the microfibers. Figure 3C reveals that the drug was released in a burst within the first 24 h, likely due to its hydrophilic nature and its deposition in the outer layer of the microfibers. Specifically, in all tested conditions, DOXY exhibited rapid release upon contact with water, with the majority being released in under 1 h. However, multilayered patches composed of alternating drug-loaded and drug-free layers achieved a more sustained release profile. This effect is attributed to the drug-free layers acting as semi-impermeable barriers that limit water diffusion into the inner microfiber layers, thereby delaying drug mobilization. These results suggest that, following the electrospinning process, the drug remains predominantly on the surface of the fibers rather than being embedded within their core. Consequently, fiber degradation is not required for drug release from the patch.

To finally verify this hypothesis, the wettability of the different microfiber formulations was assessed. As shown in Fig. 3D, a lower drug content was associated with decreased wettability, which in turn had a direct impact on the drug release profile.

As anticipated, due to the hydrophilic nature of the drug with a referred log P of −0.7, the results demonstrate an initial burst release of DOXY under semi-physiological conditions—an outcome generally considered suboptimal for therapeutic applications[19]. Nonetheless, this release profile serves as a control reference for evaluating whether the nanoparticle-based delivery strategy can achieve a more sustained and clinically advantageous release.

**Fig. 4:**
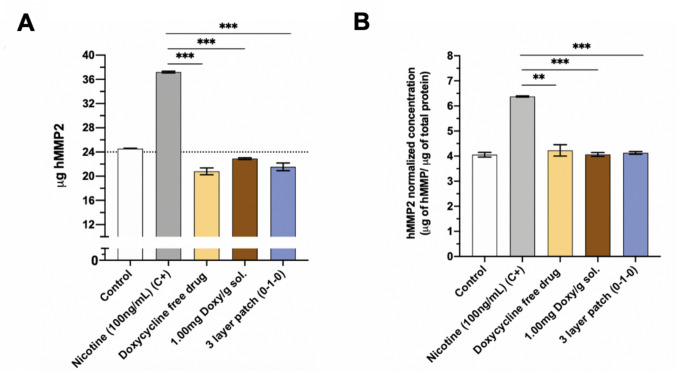
ELISA-based evaluation of the efficacy of DOXY released from the microfibers on MMP-2 mitigation after 6 days of treatment. **A** – Absolute MMP-2 concentration after application of different treatments starved with Nicotine. **B** – Normalized MMP-2 concentration values from graph A adjusted to total protein content. As *n* = 6, a parametric unpaired t-test was applied

The therapeutic efficacy of this control group in mitigating hMMP-2 expression was assessed, as shown in Fig. 4. This experiment aimed to determine whether DOXY retains its pharmacological activity after incorporation into microfibers via the electrospinning process. A loss of activity of DOXY during processing would result in diminished or absent MMP-2 inhibition, even if the drug were released. Only conditions expected to yield therapeutically relevant doses were tested, with dosing adjusted according to patch configuration—specifically, 1.0 mg single-layer and triple-layer formats. As shown in Fig. 4, the drug released from the electrospun fibers retained comparable efficacy to that of the control group, with no statistically significant differences observed when comparing positive control with other groups.

### DOXY can be efficiently encapsulated in OM-PLGA NPs

Once the cellular models and appropriate controls were fully established and validated, the therapeutic strategy involving the incorporation of DOXY into Oligopeptide-Modified Poly(lactide-co-glycolic acid) (OM-PLGA) polymeric nanoparticles (NPs) was implemented in order to retain NPs for longer time-periods. These NPs were subsequently anchored to the previously developed electrospun PCL microfibers (0.00 g DOXY/gram of organic solution).

The overall approach involved the synthesis and characterization of the NPs, followed by their integration into the surface of the polymeric microfibers. This was achieved by treating the PCL fibers with cold oxygen plasma to generate surface free radicals and then immersing them in a NP suspension to facilitate covalent attachment through chemical bonding.

The initial steps focused on the preparation of OM-PLGA NPs using a W_1_/O/W_2_double emulsion method, as previously described in the literature [20, 21]. Functionalization was achieved by conjugating the terminal ends of PLGA with the CRRR peptide, as detailed in Supplementary Information (ESI-2) and described in a previous literature [22, 23]. This strategy was selected based on the group’s prior expertise in using proprietary cationic oligopeptides to functionalize biodegradable polymers, thereby enhancing NP interaction with anionic plasma membranes and promoting intracellular delivery of therapeutic cargo [24–26].

**Fig. 5:**
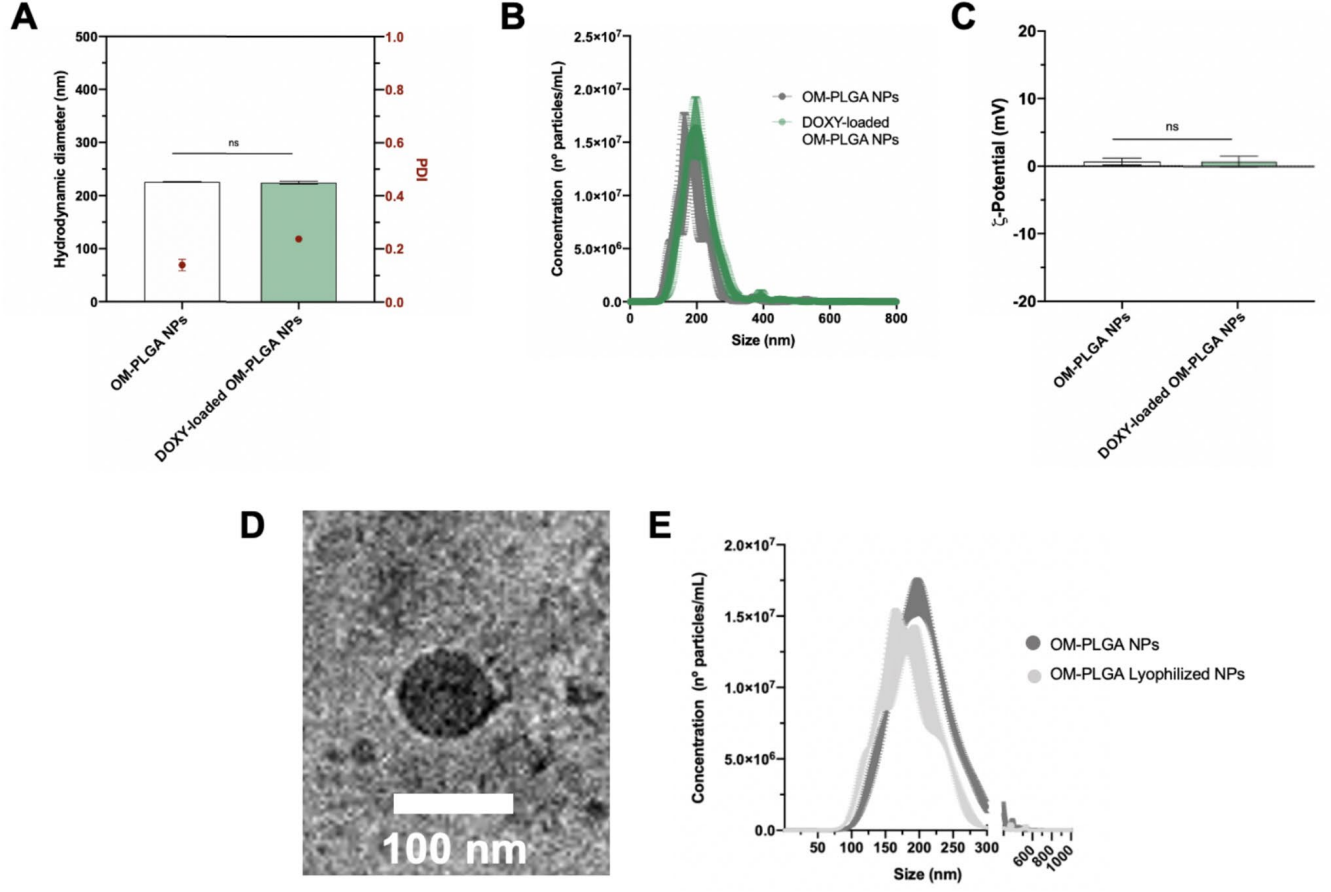
NPs characterization. **A** – Hydrodynamic diameter and Polydispersity Index characterization through DLS. **B** – Size and concentration characterization through NTA. **C** – Surface charge characterization by means of Z-potential obtained by DLS. **D** – NPs caption through Cryo-TEM. **E** – Size and concentration characterization through NTA after 2 months reconstitution

A comprehensive physicochemical characterization of the NP formulations was conducted, together with that of template nano-emulsions (see ESI-3). As shown in Fig. 5A, both drug-loaded and unloaded NPs exhibited a uniform hydrodynamic diameter 204.0 ± 3.7 nm, with a polydispersity index (PDI) under 0.25; values known to favor cellular uptake and ensure colloidal stability. These measurements, initially obtained via dynamic light scattering (DLS), were further validated by nanoparticle tracking analysis (NTA), as shown in Fig. 5B. Both techniques confirmed the same results.

Surface charge analysis revealed a slightly positive zeta potential (1.02 ± 0.7 mV), attributed to the presence of the terminal cationic peptide at the acidic end of the polymer chain (Fig. 5C), as compared to non-peptide funcationalized PLGA NPs (see ESI-4). Cryo-TEM confirmed the NPs’ spherical morphology and monodispersity (Fig. 5D) with lower diameter due to vitrification and the absence of free hydration that cause a slight contraction of the hydrated corona or surface layer of the NPs.These results are consistent with previous studies, such as Fran et al. which reported similar characteristics for DOXY-loaded nanocarriers and the ones reported in our previous paper [27, 28].

Encapsulation efficiency obtained for DOXY loaded PLGA NPs was 87.6 ± 0.6%, significantly outperforming previously reported values of approximately 60%, such as those described by Singh Y. et al*.* [29], using ultrafine PLGA NPs formulated by using double emulsion diffusion technique. Importantly, the formulated NPs retained their structural integrity following lyophilization, underscoring the robustness of the formulation and its suitability for long-term storage. Physicochemical stability was preserved for at least two months under standard storage conditions (Fig. 5F).

### DOXY-loaded NPs are safely and efficiently uptaken by cells

**Fig. 6:**
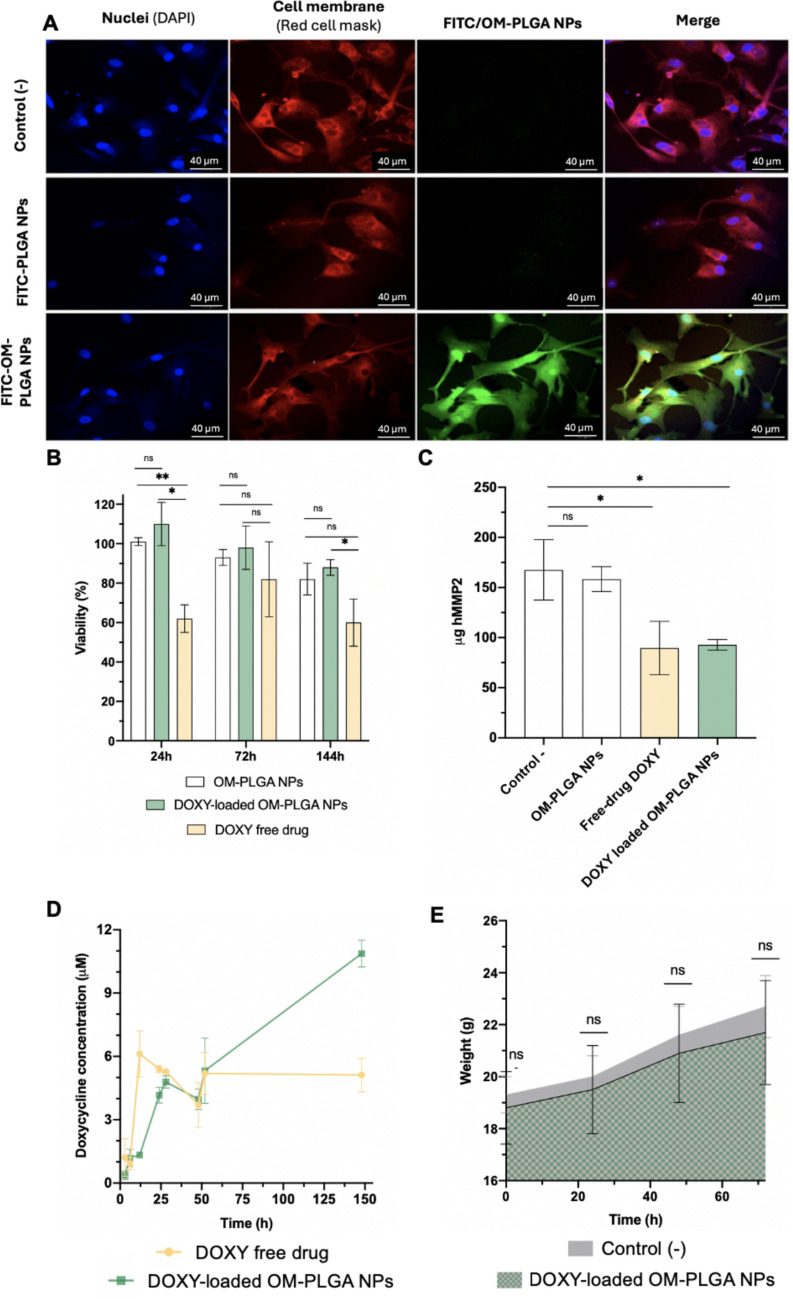
**A** – Uptake characterization through confocal microscopy of OM-PLGA NPs and FITC-OM-PLGA NPs. **B** – Viability assay of OM-PLGA NPs, DOXY loaded OM-PLGA NPs and free DOXY. **C** – Efficacy assay through hMMP-2 quantification ELISA of the different previous conditions, **D** – DOXY quantification through UHPLC-MSMS uptaken by cells and finally **E** – In vivocytotoxicity evaluation of DOXY-loaded OM-PLGA NPs

To investigate the interaction of DOXY-loaded NPs with target cells, OM-PLGA was labeled with FITC as described and incubated with HAoSMCs over various timepoints. Fluorescence intensity was subsequently analyzed (see ESI-5), revealing peak uptake at 24 h post-incubation. This confirms efficient internalization of the NPs by HAoSMCs, likely facilitated by their slightly positive surface charge (Fig. 6A) thereby enhancing intracellular delivery of DOXY.

Moreover, DOXY-loaded PLGA NPs formulation demonstrates excellent biocompatibility during more than 6 days at its effective dose and efficiently downregulates MMP-2 expression (Fig. 6B and C. In this sense, NPs demonstrate protection from intrinsic cytotoxic effect of DOXY as at all timepoints significant differences were observed when comparing to free drug. In terms of efficacy DOXY-loaded NPs are as effective as free drug in mitigating MMP-2 in AAA in vitro models.

As published before by our group, quantitative intracellular drug profiling revealed a clear advantage of the cationic NP-based delivery system over the free drug [26]. While free DOXY exhibited rapid uptake via passive diffusion, its intracellular concentration declined sharply over time, reflecting limited retention and suboptimal pharmacokinetics. In contrast, DOXY-loaded nanoparticles, internalized predominantly through active endocytic pathways, achieved a markedly more sustained intracellular presence. This extended retention, as shown in the time-course analysis (Fig. 6D), underscores the ability of the NP system to overcome rapid efflux and degradation typically associated with free DOXY. These findings strongly support the use of NPs-mediated delivery as a superior strategy for achieving prolonged therapeutic activity at the cellular level.

Given the excellent biocompatibility and therapeutic efficacy demonstrated by the DOXY-loaded OM-PLGA system, preliminary in vivo safety studies were conducted to further assess its translational potential. Notably, the results revealed that even at doses tenfold higher than the equivalent therapeutic systemic dose, no signs of cytotoxicity or adverse effects were observed. These findings **(**Fig. 6E**)** underscore the safety profile of the formulation and its suitability for future clinical development.

### Embedding DOXY-loaded NPs in polymeric microfibers as a local delivery system

Following the independent optimization of both systems, DOXY-loaded NPs were successfully integrated into PCL microfibers. To enhance surface activation and promote effective NPs anchoring, the microfibers underwent cold plasma treatment using oxygen gas. This treatment generated reactive free radicals on the PCL surface, thereby increasing surface energy and introducing functional groups conducive to NP binding. Immediately after plasma activation, the fibers were immersed in an aqueous dispersion of DOXY-loaded NPs to facilitate their adsorption onto the microfiber surface for 2 h in constant shaking (200 rpm) at RT.

**Fig. 7:**
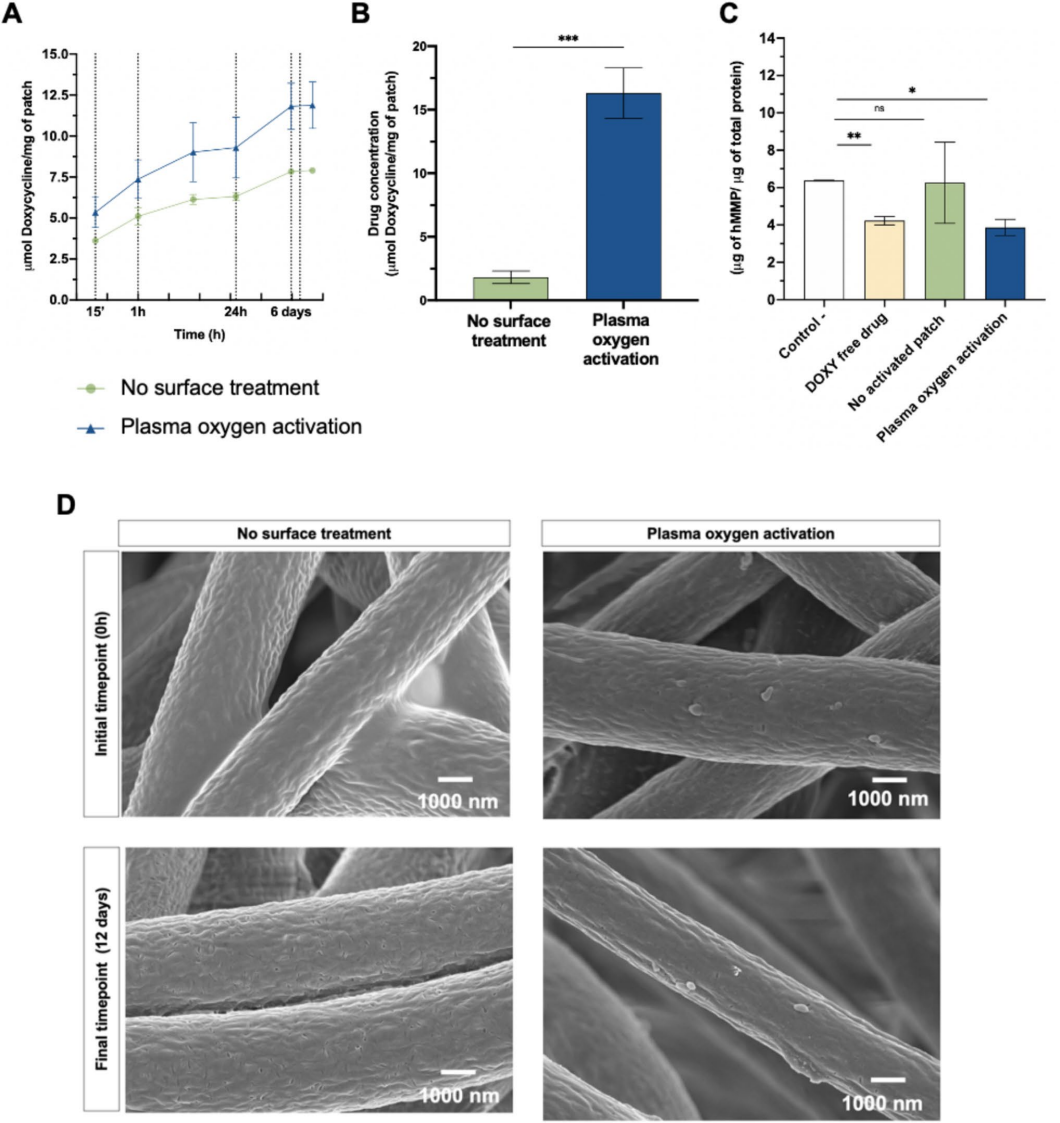
Nanoparticles anchoring through oxygen cold-plasma surface treatment of the microfibers. **A** – DOXY release profile from the microfibers comparing surface treatment and non-surface treatment conditions. **B** – Encapsulation efficiency comparison between activated and non-activated microfibers. **C** – MMP-2 concentration evaluation after different treatments 6 days after exposure through ELISA technique. And **D** – Visual imaging of the microfibers in the different conditions. As *n *= 6, a parametric unpaired t-test was applied in Fig. 3C

FESEM confirmed the stable and homogeneous attachment of NPs to the microfiber surface, with strong interfacial interactions maintained even after extended incubation periods (Fig. 7D**)**. This stability is presumed to result from covalent or semi-covalent bonding between the NPs and the plasma-treated fiber surface, supporting further investigation into the drug release behavior.

To evaluate sustained release performance and confirm the need of microfibers activation for the semi-covalent NP attachment, standardized round 10 cm^2^ patches composed of PCL microfibers were prepared under two conditions: with and without plasma-treated microfibers. Both type of samples were incubated in DOXY-loaded NP suspensions under identical conditions. First studies demonstrate more than a fivefold increase in NP loading when activating microfibers patches with plasma, resulting in significantly enhanced and prolonged DOXY release profiles (Fig. 7C) over a 12-day period revealing a cumulative release.

Importantly, functional assays evaluating MMP-2 expression showed that only activated microfibers achieved therapeutically relevant drug concentrations, effectively suppressing MMP-2 activity. In contrast, non-activated microfibers failed to elicit a comparable biological response. This is likely attributable to its limited NP adsorption capacity and subtherapeutic release profile. These findings highlight the critical importance of plasma surface modification in enabling the therapeutic efficacy of microfiber-based drug delivery platforms.

In addition to evaluating the modulation of MMP activity, we examined how DOXY, delivered either as free drug or encapsulated within OM-PLGA NPs, impacts the structural organization of extracellular type I collagen secreted by HAoSMCs. When cultured for more than eight days in the presence of ascorbic acid, as described before by López-Guimet et al. [30], these cells assemble a characteristic fibrillar collagen network, which serves as a relevant readout of ECM integrity. Exposure to nicotine (100 ng/mL), used to mimic AAA-like pathological conditions, profoundly disrupted collagen architecture. Under these conditions, type I collagen appeared markedly reduced and lost its extended fibrillar organization, consistent with ECM degradation typically observed in aneurysmal tissue.

**Fig. 8:**
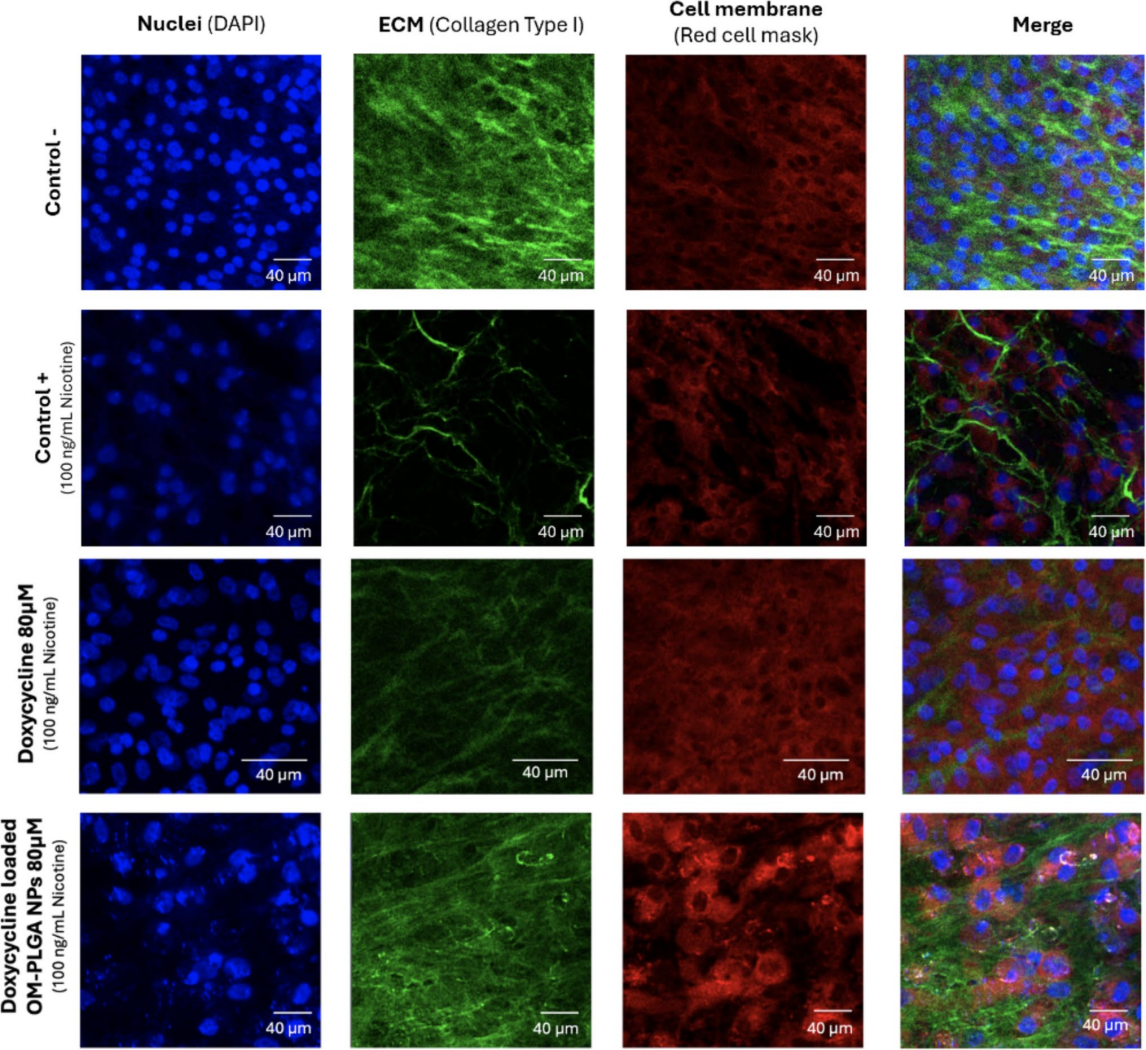
Effects of free DOXY and DOXY-loaded OM-PLGA nanoparticles on collagen type I in an in-vitro AAA model. Confocal fluorescence microscopy of untreated cells (Control −, no nicotine), and in vitro AAA model cells generated by exposure to nicotine (Control +). The AAA model was further treated either with free doxycycline (DOX 80 µM) or with DOX-loaded OM-PLGA nanoparticles. Nuclei were stained with DAPI (blue), collagen type I with FITC-conjugated antibody (green), and cell membranes with Red Cell Mask (red), last column corresponds to merge. Scale bar: 40 µm. Extra. Images found in Figure S1

As shown in Fig. 8, treatment with free DOXY (80 µM for 4 days) partially restored the fibrillar morphology, yielding a denser and more continuous collagen mesh. Importantly, DOXY-loaded OM-PLGA NPs elicited a similar restorative effect, promoting a more homogeneous and extended three-dimensional collagen fibrillar network despite the presence of nicotine.

These findings indicate that both free DOX and NP-delivered DOX attenuate nicotine-induced ECM degradation, supporting their potential to preserve collagen integrity in AAA-mimicking environments.

## Conclusions

In this study, we present for the first time a novel strategy aimed at enhancing the therapeutic potential of DOXY in AAA treatment based on the main extracellular degrading enzyme, MMP-2. DOXY encapsulation into OM-PLGA NPs was achieved using a double emulsion solvent evaporation technique followed by a low-energy phase inversion composition template method, yielding NPs with high encapsulation efficiency, low cytotoxicity, sustained drug release profiles, and promising therapeutic performance in MMP-2 mitigation in AAA condition. Furthermore, we introduced a novel surface modification technique using cold plasma treatment to covalently anchor DOXY-loaded OM-PLGA NPs onto PCL biodegradable microfibers, as local release platform. Thus, enabling a progressive, efficacious and localized drug release for higher control and long-term mitigation of MMP-2 and collagen type I degradation. When comparing this strategy with DOXY embedded microfibers, NPs shows markedly higher efficacy as it is possible to control DOXY release in time. Altogether, our findings provide new insights into the design of NP-based drug delivery systems and support their potential for targeted vascular therapies.

## Supplementary Information

Below is the link to the electronic supplementary material.

Supplementary file1 (DOCX 14989 KB)

## Data Availability

Data will be made available upon reasonable request to corresponding authors.

## References

[1] Renna NF, De Las Heras N and Miatello RM Pathophysiology of vascular remodeling in hypertension. Int. J. Hypertens. 2013. 10.1155/2013/808353. 10.1155/2013/808353 PMC3736482 23970958

[2] Laronha H, Caldeira J. Structure and function of human matrix metalloproteinases. Cells. 2020. 10.3390/cells9051076. 32357580 10.3390/cells9051076 PMC7290392

[3] Livesay JJ, Messner GN, Vaughn WK. Milestones in the treatment of aortic aneurysm: Denton A. Cooley, MD, and the Texas Heart Institute. Tex Heart Inst J. 2005;32:130–4. 16107099 PMC1163455

[4] Golledge J, Shi GP, Norman P. Pathogenesis of aortic aneurysms. Mech Vasc Dis A Ref B Vasc Spec. 2011;33:227–46. 30485032

[5] Norman PE, Powell JT. Abdominal aortic aneurysm: the prognosis in women is worse than in men. Circulation. 2007;115:2865–9. 17548742 10.1161/CIRCULATIONAHA.106.671859

[6] Karthikesalingam A, et al. Mortality from ruptured abdominal aortic aneurysms: clinical lessons from a comparison of outcomes in England and the USA. Lancet. 2014;383:963–9. 24629298 10.1016/S0140-6736(14)60109-4

[7] Steuer J, Lachat M, Veith FJ, Wanhainen A. Endovascular grafts for abdominal aortic aneurysm. Eur Heart J. 2016;37:145–51. 26543044 10.1093/eurheartj/ehv593

[8] Cerofolini L, Fragai M, Luchinat C. Mechanism and inhibition of matrix metalloproteinases. Curr Med Chem. 2018;26:2609–33. 10.2174/0929867325666180326163523 29589527

[9] Dosta P, et al. Delivery of anti-microRNA-712 to inflamed endothelial cells using poly(β-amino ester) nanoparticles conjugated with VCAM-1 targeting peptide. Adv Healthc Mater. 2021;10:1–21. 10.1002/adhm.202001894 PMC8277885 33448151

[10] Balà N, Aranda A, Teixidó P, Molhoek C, Moreno-Jiménez I, Febas G, et al. In vivo efficacy of an adhesive bioresorbable patch to treat aortic dissections. JACC Basic Transl Sci. 2024;9(1):65–77. 10.1016/j.jacbts.2023.08.002. 10.1016/j.jacbts.2023.08.002 PMC10864981 38362347

[11] Fornaguera C, Calderó G, Solans C. Electrolytes as a tuning parameter to control nano-emulsion and nanoparticle size. RSC Adv. 2016;6:58203–11.

[12] Dobrovolskaia, et al. Method for analysis of nanoparticle hemolytic properties in vitro. Nano Lett. 2008;8:2180–7. 18605701 10.1021/nl0805615 PMC2613576

[13] Dobrovolskaia MA and McNeil SE Handbook of immunological properties of engineered nanoparticles. (SAIC-Frederick Inc., 2013). https://d1wqtxts1xzle7.cloudfront.net/107897420/43616d4e36d358ca4b884345283de8f8892e-libre.pdf?1701073625=&response-contentdisposition=inline%3B+filename%3DHandbook_of_Immunological_Properties_of.pdf&Expires=1765037234&Signature=Gku5j1j94Fr0owKLM~VZTwrTTZkVRFlTsDPzxFVpZuB9zza6m4romjzm1ScmmOQ4pGAxWT-KtR-gizoswaMf5MwGGS98YW4R87yoOJBJ6IWiZl4GdKqy17Gx8y45mJCG6vhaNdqfuHxKDtIkVRNmz0O5gck31fRKjr3w2Ta5wXinCm6y44amXCt~HPoMUc3J7BsfCJj5BvvQXG0y-Bu2fFBB68ec~DcrST4vzKa4gpTJnpGnHJPUqBIPYWQEaSEyVTe-SS3hKIW75rMzu5mDinQGD8f2mZyUcRsDI5RrdwtamR4l7QuRF2OyLLGdEoT4A__&Key-Pair-Id=APKAJLOHF5GGSLRBV4ZA

[14] McNeil SE Characterization of nanoparticles intended for drug delivery. 2011. https://ndl.ethernet.edu.et/bitstream/123456789/39011/1/Scott%20E.%20McNeil.pdf

[15] O’Brien RW, White LR. Electrophoretic mobility of a spherical colloidal particle. J Chem Soc Faraday Trans 2. 1978;74:1607.

[16] Cabral-Pacheco GA, et al. The roles of matrix metalloproteinases and their inhibitors in human diseases. Int J Mol Sci. 2020;21:1–53. 10.3390/ijms21249739 PMC7767220 33419373

[17] Šišková AO, et al. The drug-loaded electrospun poly(ε-caprolactone) mats for therapeutic application. Nanomaterials. 2021. 10.3390/nano11040922. 33916638 10.3390/nano11040922 PMC8066245

[18] Behnoosh Zarei, Mohammad Akrami, Niloufar Rezaei, Mohammad Mahdavi, Mojtaba Kamankesh, Ismaeil Haririan, Mehdi Asadi, M. N.-N. A doxycycline-loaded microfiber of poly-metformin/PCL for eradicating melanoma stem cells,. Int. J. Pharm. 2024;600 10.1016/j.ijpharm.2024.124358 38897492

[19] Doxycycline, D. Dom - DOXYCYCLINE. 2006; 1–27. https://www.uspnf.com/sites/default/files/usp_pdf/ES/rb/doxycycline-hyclate-tabs-rb-m28370-spa.pdf

[20] Sadurní N, Solans C, Azemar N, García-Celma MJ. Studies on the formation of O/W nano-emulsions, by low-energy emulsification methods, suitable for pharmaceutical applications. Eur J Pharm Sci. 2005;26:438–45. 10.1016/j.ejps.2005.08.001. 10.1016/j.ejps.2005.08.001 16153811

[21] Bofill-Bonet C, et al. Fine-tuning formulation and biological interaction of doxorubicin-loaded polymeric nanoparticles via electrolyte concentration modulation. J Mol Liq. 2023. 10.1016/j.molliq.2023.122986.

[22] Pinto MSL, et al. Polymer-based coating of adeno-associated viral particles as a new strategy to evade immune response for DMD treatment. J Control Release. 2025. 10.1016/j.jconrel.2025.113896. 40441494 10.1016/j.jconrel.2025.113896

[23] Futaki S, et al. Arginine-rich peptides An abundant source of membrane-permeable peptides having potential as carriers for intracellular protein delivery. J Biol Chem. 2001;276:5836–40. 11084031 10.1074/jbc.M007540200

[24] Fornaguera C, et al. Engineering oncogene-targeted anisamide-functionalized pBAE nanoparticles as efficient lung cancer antisense therapies. RSC Adv. 2023;13:29986–30001. 37842686 10.1039/d3ra05830a PMC10573942

[25] Fornaguera C, et al. In vivo retargeting of poly(beta aminoester) (OM-PBAE) nanoparticles is influenced by protein corona. Adv Healthc Mater. 2019;8:1–11. 10.1002/adhm.201900849 31478348

[26] Navalón-López M, Dols-Perez A, Grijalvo S, Fornaguera C, Borrós S. Unravelling the role of individual components in pBAE/polynucleotide polyplexes in the synthesis of tailored carriers for specific applications: on the road to rational formulations. Nanoscale Adv. 2023;5:1611–23. 36926558 10.1039/d2na00800a PMC10012844

[27] Feiner-Gracia N, et al. Cell penetrating peptide grafting of PLGA nanoparticles to enhance cell uptake. Eur Polym J. 2018;108:429–38.

[28] Misra R, Sahoo SK. Antibacterial activity of doxycycline-loaded nanoparticles. In: Methods in Enzymology, vol. 509. Elsevier Inc.; 2012. 10.1016/B978-0-12-391858-1.00004-6 22568901

[29] Singh Y, et al. Subcutaneously administered ultrafine PLGA nanoparticles containing doxycycline hydrochloride target lymphatic filarial parasites. Mol Pharm. 2016;13:2084–94. 27144397 10.1021/acs.molpharmaceut.6b00206

[30] López-guimet J, Andilla J, Loza-alvarez P, Egea G. High-resolution morphological approach to analyse elastic laminae injuries of the ascending aorta in a murine model of Marfan syndrome. Sci Rep. 2017(1). 10.1038/s41598-017-01620-8. 10.1038/s41598-017-01620-8 PMC5431420 28473723

