# Supplementary material for: Localized doxycycline delivery via polymeric nanoparticles anchored to electrospun PCL microfiber patch for MMP-2 inhibition in abdominal aortic aneurysm
Source: Drug Deliv Transl Res. 2026 Jan 20;16(8):2807–23. doi: 10.1007/s13346-025-02027-z (PMC13346288; doi:10.1007/s13346-025-02027-z)

**Localized Doxycycline Delivery via Polymeric Nanoparticles Anchored to Electrospun Microfiber Patch for MMP-2 Inhibition in Abdominal Aortic Aneurysm**

*Carles Bofill-Bonet^1^, German Febas^2^, Margalida Artigues^1^, Inés Moreno-Jiménez^2^, Noemí Balà^2^, Jordi Martorell^1,2^, Salvador Borrós^1,2*^, Cristina Fornaguera^1*^.*

*^1^ Institut Químic de Sarrià (IQS), Universitat Ramon Llull (URL), Via Augusta 390, 08017, Barcelona, Spain*

*^2^ Aortyx SL, Teia, Spain*

*Correspondence to:* [*salvador.borros@iqs.url.edu*](mailto:salvador.borros@iqs.url.edu) *and* [*cristina.fornaguera@iqs.url.edu*](mailto:cristina.fornaguera@iqs.url.edu)

**Electronic Supplementary Information (ESI):**

**ESI – 1 (Electrospinning used conditions table):**

**Table 1.** Optimized parameters for each sheet of formulated microfibers.

| **Sheet int. reference** | **Doxy (mg/g)** | **Time (min)** | **Distance (cm)** | **Volume (mL)** | **Voltage (kV)** | **Observation** |
| --- | --- | --- | --- | --- | --- | --- |
| **D001** | 0 | nt150 | 22 | 2.5 | 18.0 | Good stability. 26.5ºC/45%RH |
| **D002** | 0.5 | 135 | 20 | 2.5 | 19.1 | Little fluctuation. 24ºC/49%RH |
| **D003** | 0.5 | 150 | 21 | 2.5 | 14.9 | Jet stable.  23.5ºC/57%RH |
| **D004** | 0.5 | 150 | 21 | 2.5 | 17.5 | Slight pulsatility. 23ºC/53%RH |
| **D005** | 1.0 | 150 | 21 | 2.5 | 22.3 | Stable jet. 25ºC/45%RH |
| **D006** | 1.0 | 150 | 21 | 2.5 | 17.5 | Slight pulsatility. 23ºC/53%RH |
| **D007** | 1.0 | 150 | 22 | 2.5 | 19.4 | Stable jet. 25ºC/35%RH |
| **D008** | 0.5 | 15 | 21 | 0.25 | 16.9 | Stable jet. 23.7ºC/50%RH |
| **D009** | 0.5 | 30 | 21 | 0.50 | 17.4 | Small initial drops.  24.2ºC/44%RH |
| **D010** | 0.5 | 45 | 21 | 0.75 | 17.5 | Stable jet. 25.7ºC/40%RH |
| **D011** | 1.0 | 15 | 21 | 0.25 | 18.2 | Stable jet. 23.5ºC/57%RH |
| **D012** | 1.0 | 30 | 21 | 0.50 | 18.7 | Slight pulsatility.  23.5ºC/57%RH |
| **D013** | 1.0 | 45 | 21 | 0.75 | 22.1 | Stable jet. 23.5ºC/57%RH |
| **D014** | 0.1 | 90 | 21 | 1.5 | 23.2 | Great stability.  26.5ºC /45%RH |
| **D015** | 0.25 | 90 | 21 | 1.5 | 22.8 | Stable jet. 26ºC/53%RH |
| **D016** | 1-0-1 | 30x3 | 21 | 1.5 | 14.9 | Stable jet. 23.5ºC/57%RH |
| **D017** | 1-0-1-0 | 22.5x4 | 21 | 1.5 | 23.6 | Slight pulsatility. 23ºC/53%RH |

**ESI – 2 (OM-PLGA characterization):**


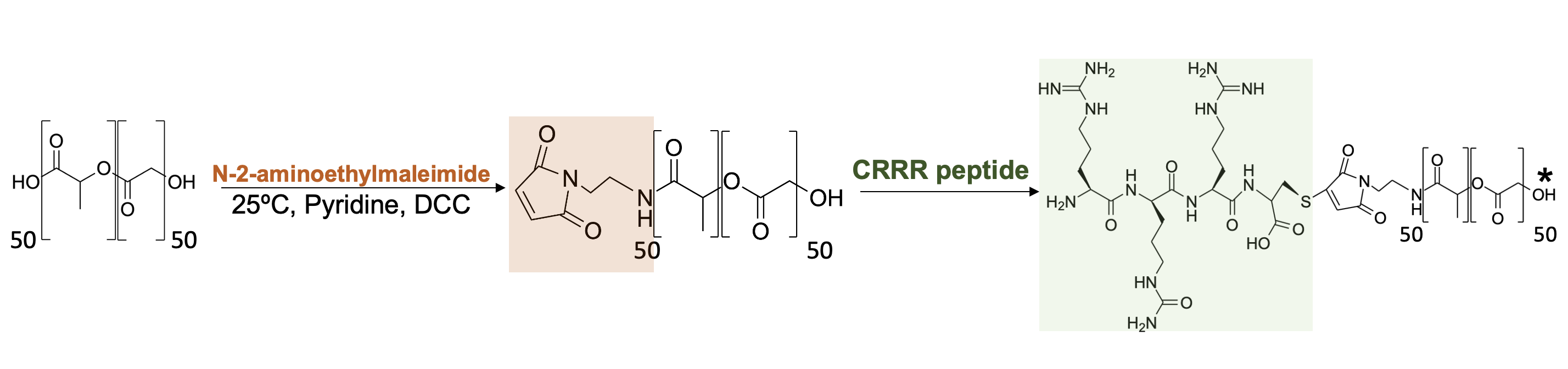


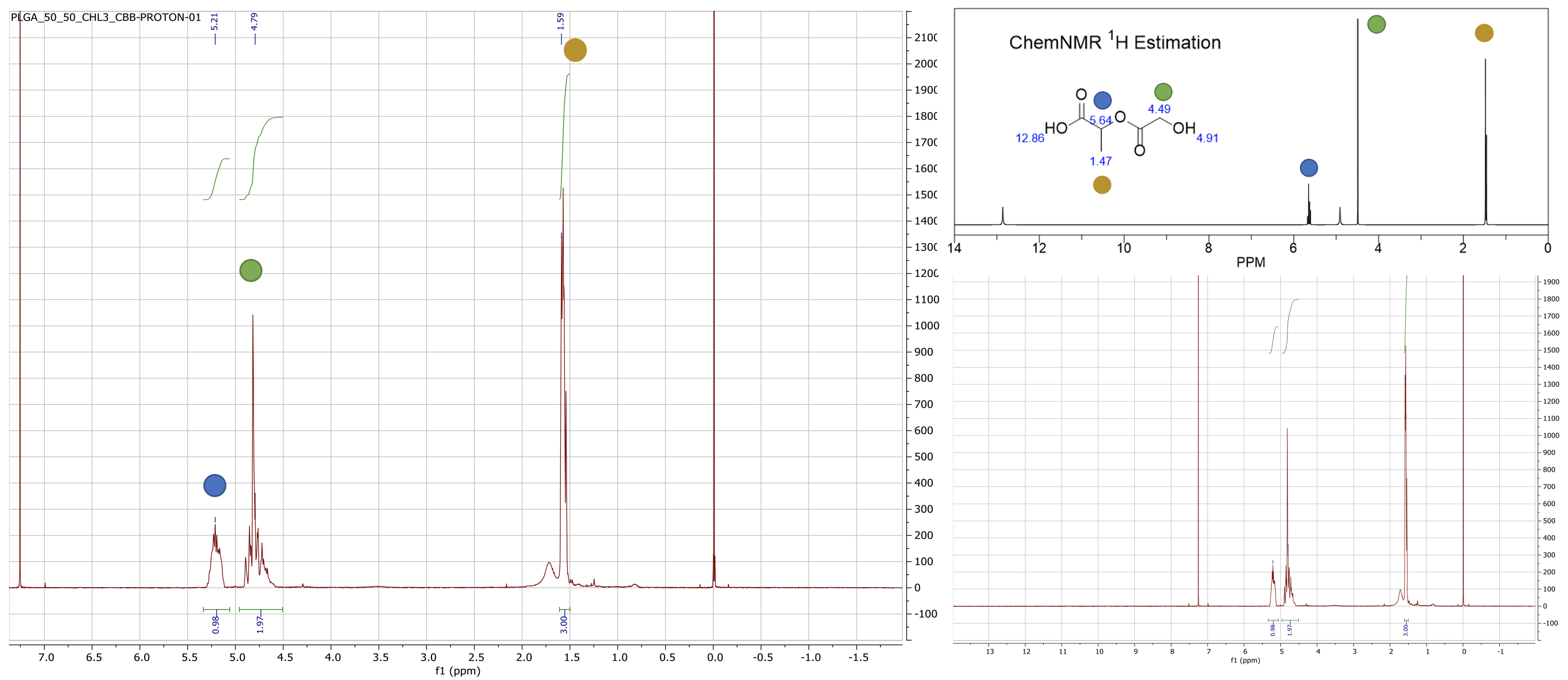


The ¹H-NMR spectrum of PLGA (CDCl₃, 400 MHz) showed characteristic signals at δ 5.2 ppm (m, CH of lactic acid units), 4.8 ppm (m, CH₂ of glycolic acid units), and 1.6 ppm (d, CH₃ of lactic acid units). The chemical shifts are consistent with the expected structure of PLGA, confirming the presence of both lactic and glycolic components. The integration of the CH (δ 5.2 ppm), CH₂ (δ 4.8 ppm), and CH₃ (δ 1.6 ppm) signals allows the estimation of the lactic-to-glycolic acid ratio in the copolymer.


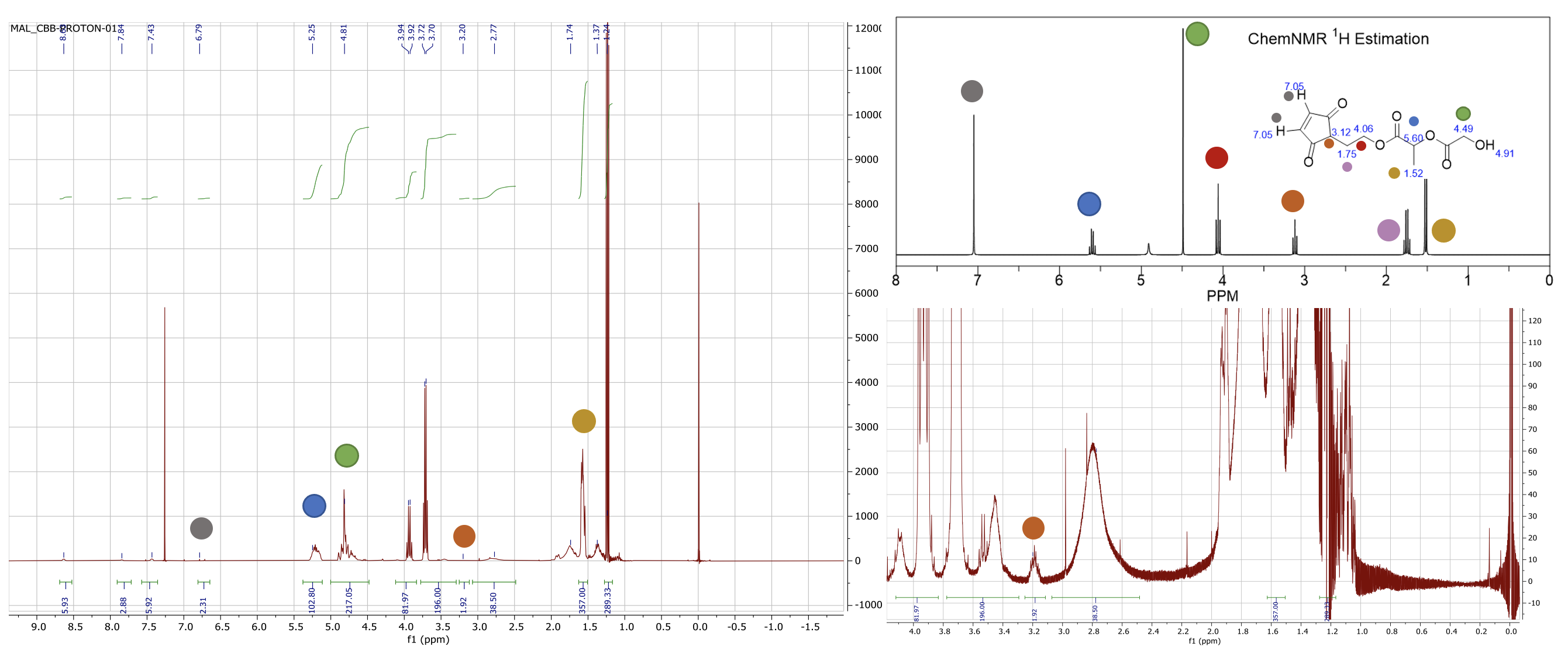


The ¹H-NMR spectrum of PLGA-MAL (CDCl₃, 400 MHz) displayed the characteristic signals of PLGA at δ 5.2 ppm (m, CH of lactic acid units), 4.8 ppm (m, CH₂ of glycolic acid units), and 1.6 ppm (d, CH₃ of lactic acid units). In addition, new resonances attributable to the maleimide moiety were observed at δ 6.7–7.0 ppm (s, CH=CH of the maleimide ring), confirming the successful conjugation. Aliphatic methylene groups from the linker appeared between δ 3.5 and 4.2 ppm, while additional weak signals around δ 1.8–2.1 ppm correspond to the spacer’s aliphatic chains. The appearance of the maleimide-specific peaks together with the preservation of the PLGA signals confirmed the successful functionalization.


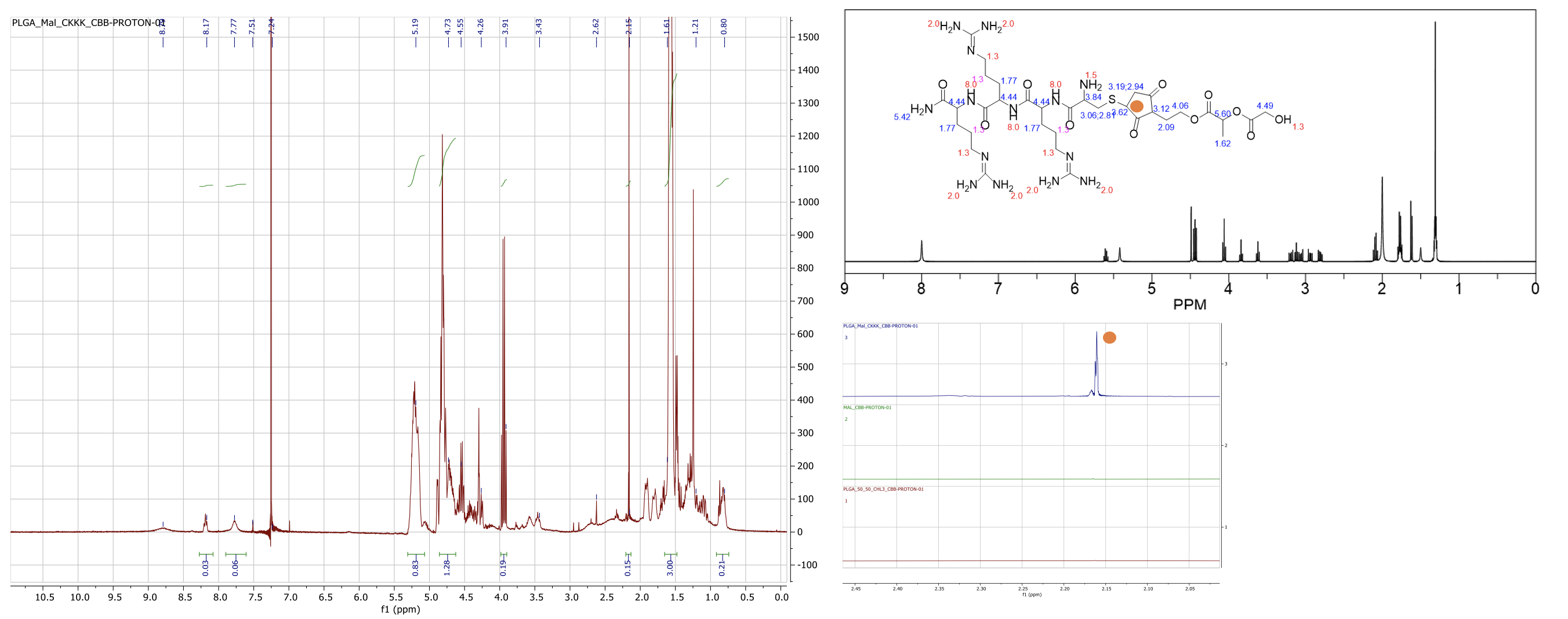


The ¹H-NMR spectrum of PLGA-MAL-CRRR (DMSO‑d₆, 400 MHz) retained the characteristic PLGA signals at δ ~5.2 ppm (m, CH of lactic units), 4.8 ppm (m, CH₂ of glycolic units), and 1.6 ppm (d, CH₃ of lactic units). The maleimide proton signals previously observed at δ ~6.7–7.0 ppm significantly decreased/disappeared, consistent with the Michael-type addition of the cysteine thiol group to the maleimide ring. New resonances attributable to the peptide were observed: broad multiplets between δ 3.0 and 4.0 ppm (CH and CH₂ of arginine side chains), and characteristic aliphatic signals at δ 1.4–1.8 ppm. The appearance of these peptide-specific peaks, together with the loss of maleimide unsaturation signals, confirmed the successful conjugation of the CRRR peptide to PLGA-MAL. Reaction efficiency was reported at 78%.

**ESI – 3 (Nanoemulsion template characterization):**


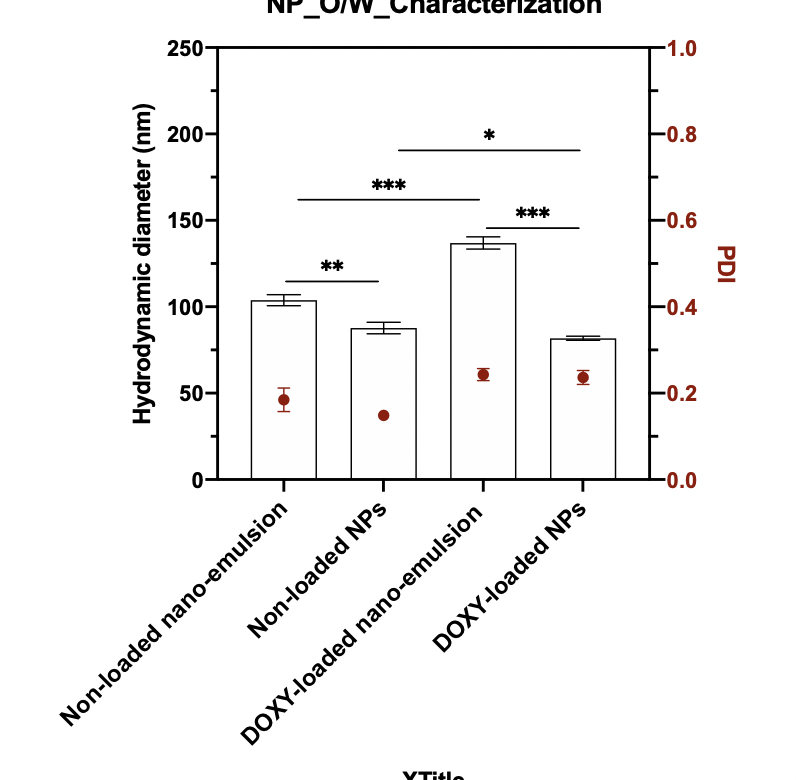


**ESI – 4 (Surface charge comparison):**


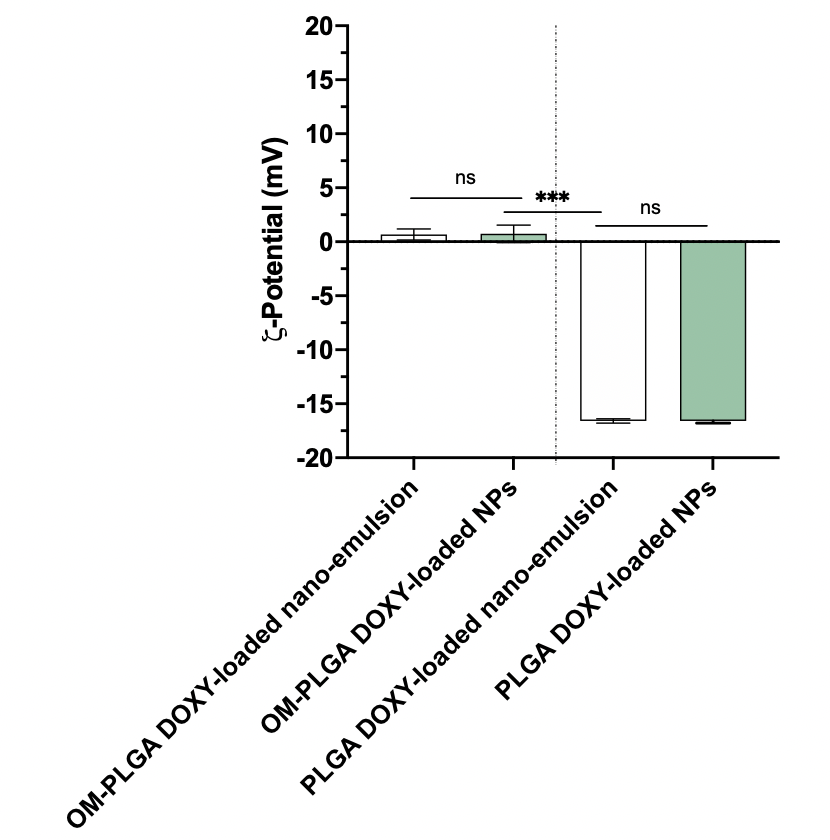


**ESI – 5 (FITC-PLGA Nanoparticles uptake characterization):**


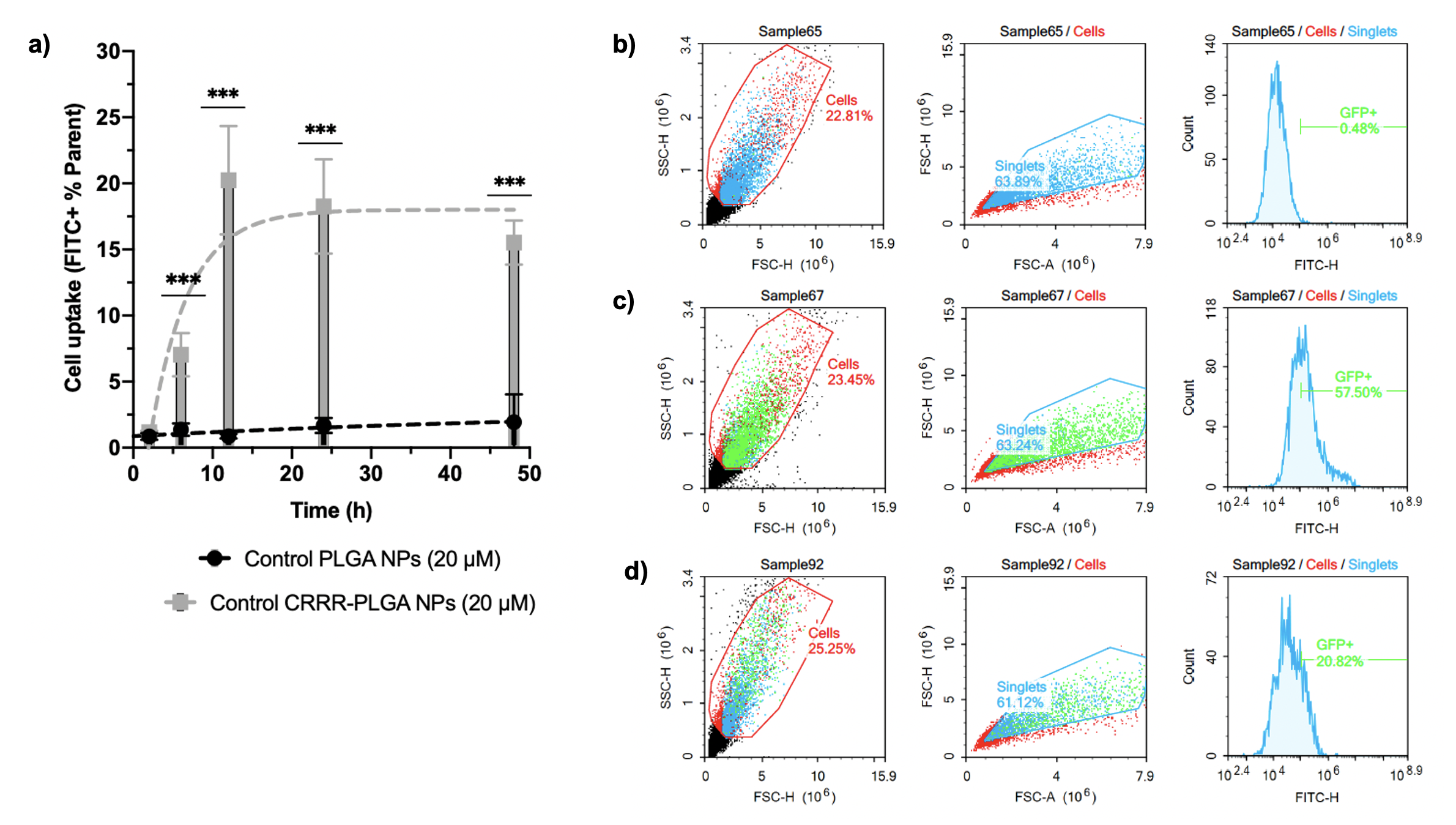

Supplement: Supplementary file 1 — Supplementary file1 (DOCX 14989 KB) [file 13346_2025_2027_MOESM1_ESM.docx]
